# *otargen*: GraphQL-based R package for tidy data accessing and processing from Open Targets Genetics

**DOI:** 10.1093/bioinformatics/btad441

**Published:** 2023-07-19

**Authors:** Amir Feizi, Kamalika Ray

**Affiliations:** Bioinformatics Department, OMass Therapeutics, Oxford Business Park, ARC, Oxford OX4 2GX, United Kingdom; Bioinformatics Department, OMass Therapeutics, Oxford Business Park, ARC, Oxford OX4 2GX, United Kingdom

## Abstract

**Motivation:**

Open Target Genetics is a comprehensive resource portal that offers variant-centric statistical evidence, enabling the prioritization of causal variants and the identification of potential drug targets. The portal uses GraphQL technology for efficient data query and provides endpoints for programmatic access for R and Python users. However, leveraging GraphQL for data retrieval can be challenging, time-consuming, and repetitive, requiring familiarity with the GraphQL query language and processing outputs in nested JSON (JavaScript Object Notation) format into tidy data tables. Therefore, developing open-source tools are required to simplify data retrieval processes to integrate valuable genetic information into data-driven target discovery pipelines seamlessly.

**Results:**

*otargen* is an open-source R package designed to make data retrieval and analysis from the Open Target Genetics portal as simple as possible for R users. The package offers a suite of functions covering all query types, allowing streamlined data access in a tidy table format. By executing only a single line of code, the *otargen* users avoid the repetitive scripting of complex GraphQL queries, including the post-processing steps. In addition, *otargen* contains convenient plotting functions to visualize and gain insights from complex data tables returned by several key functions.

**Availability and implementation:**

*otargen* is available at https://amirfeizi.github.io/otargen/.

## 1 Introduction

In recent years, various studies have demonstrated that approved drug targets often exhibit a higher enrichment of genes with genetic evidence linked to diseases (Carter *et al.* 2013, Nelson *et al.* 2015). Open Target Genetics (OTG) is an open-source tool to explore and prioritize potential causal variants and genes associated with human diseases and traits. Leveraging aggregated data from genome-wide association studies (GWAS) and functional genomics, OTG provides valuable insights into the genetic landscape of diseases. The GWAS data within OTG includes statistical summary data from prominent genomics projects such as UK Biobank (https://www.ukbiobank.ac.uk/), FINNGEN (https://www.finngen.fi/) (Mountjoy *et al.* 2021), and GWAS Catalog (a specific database for collecting and curating GWAS data) (https://www.ebi.ac.uk/gwas/) (Ghoussaini *et al.* 2021). The OTG is a spin-out database from the original portal of the Open Targets Platform (https://platform.opentargets.org/), a collaborative initiative between EMBL-EBI and several pharmaceutical companies to support systematic identification and prioritization of potential therapeutic drug targets. These two platforms integrate publicly available data sets, including Open Targets consortium data and genetic evidence for gene-disease associations. In addition, relevant annotation information about a target, disease, phenotype, drug, and their most relevant relationships are integrated (Ochoa *et al.* 2022).

The OTG platform supports various levels of data access. The web user interface (UI) offers a practical and user-friendly experience for low-throughput and exploratory analyses. The GraphQL API is available for medium-scale data access, providing a flexible and powerful interface for retrieving specific data subsets. Additionally, the OTG platform enables access to the complete data sets for large-scale analyses (e.g. via FTP). However, the GraphQL interface is a key advantage of OTG because it provides flexible and efficient access to underlined data. Compared to traditional REST APIs, GraphQL offers valuable benefits, including constructing queries that return only the user-defined fields and traverse the graph database to minimize the number of queries. By eliminating the need for round trips to the server, GraphQL improves query performance and reduces network overhead. Users can interact with the OTG portal’s GraphQL API using command-line tools like *curl* and *wget*, executing queries via the provided browser endpoint, or connecting from R and Python environments. While these APIs facilitate data access, they have several challenges. For instance, the browser endpoint helps explore the schema, learn GraphQL, and test queries but is unsuitable for systematic data access. Finalized queries must be transferred and executed in R or Python, leading to inefficiencies. Additionally, users often repeatedly have to write identical queries, resulting in time-consuming and redundant processes. Furthermore, the nested and complex nature of the returned data in JSON format by GraphQL queries poses difficulties in converting it into tidy data tables, a common concern among users. To address these challenges, we have developed the *otargen* R package based on the *ghql* package, which enables connecting and data transactions with OTG’s GraphQL endpoint from the R environment. The primary goal of *otargen* is to facilitate systematic access to the OTG platform, enabling efficient integration of genetic evidence with data-driven target discovery pipelines commonly used in bioinformatics and genetics research.

## 2 Description

The *otargen* package offers a wide range of functions that align with the active GraphQL queries in the OTG schema. To maintain consistency, the function names directly correspond to the query names from GraphQL query schema in OTG. In addition to data retrieval, the package includes specialized plotting functions that enhance data visualization and support insightful analysis. A dedicated function for executing custom GraphQL queries is designed to accommodate specific query requirements. In the upcoming sections, we overview the functionalities offered by *otargen*, organized into three main categories with examples.

### 2.1 Schema query functions

These functions are wrappers for different GraphQL query types defined in the OTG schema. They are specifically designed to retrieve data based on various search parameters, including *variant IDs*, *gene IDs*, and GWAS *study IDs* and perform post-processing steps and return tidy outputs. When executed, each function in this category returns a tidy data table in *tibble* format, ensuring structured and easily accessible data representation.

- *Example—*Retrieve L2G model summary data

In this exemplary usage, we demonstrate the application of the *studiesAndLeadVariantsForGeneByL2G()* function to obtain one of the core data tables provided by OTG platform. The returned results are the outputs of the OTG’s “locus-to-gene” (L2G) machine learning model, which leverages genetic and functional genomics features to prioritize likely causal genes at each GWAS locus. By specifying the gene(s) of interest and applying customized filters, the function retrieves all partial scores for lead variants in the locus of the specified gene. The resulting data table is comprehensive and presented in a tidy *tibble* format. Below is the example code snippet for this use case.


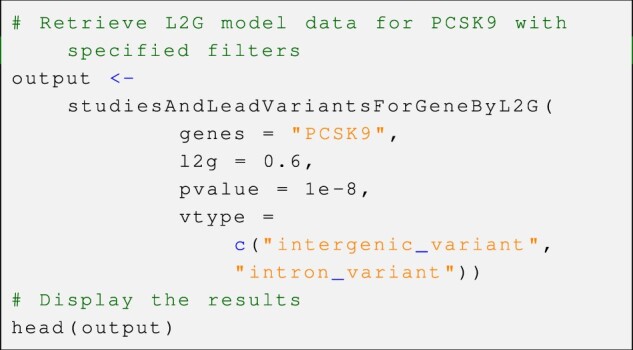


**Figure 1.**
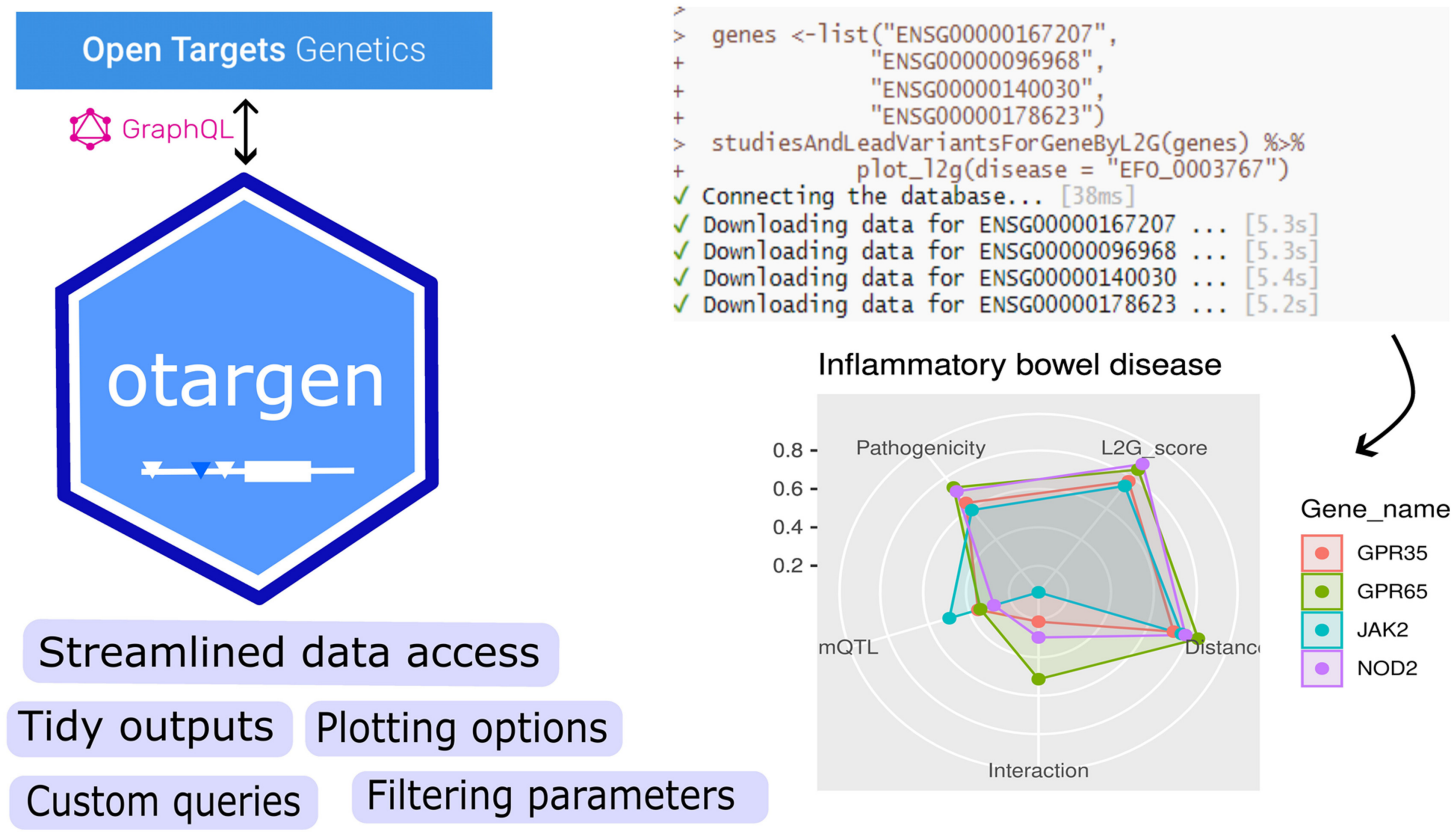
Overview of *otargen*.

As shown in Fig. 1, users can pipe the output from this function into the *plot_l2g()* and visually explore and prioritize multiple genes for a specific disease.

### 2.2 Plotting functions

*otargen* provides plotting options for several complex data tables returned by its core query functions, including PheWAS (Phenome Wide Association Study) statistics, L2G model, colocalization statistics, and GWAS summary statistics. In the below code snippet, we have shown how the user can quickly generate insightful visualizations by piping the output of such queries into the corresponding plotting function.

- *Example—*Plot data obtained from the *PheWAS()* function

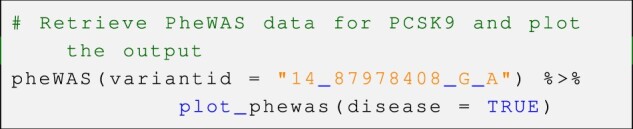


### 2.3 Custom query function

*otargen* provides an additional wrapper function *run_custom_ query()* that can execute the user’s custom queries from the OTG GraphQL endpoint. The users must provide a custom GraphQL query code as a character string and the corresponding variable list as arguments to this function.

In *otargen* package, we have extensively used diverse functionalities from *tidyverse* multi-package to convert complex nested query results in JSON format into a structured *tibble* form. We have used *devtools* package toolbox for developing *otargen* and *pkgdown* package https://pkgdown.r-lib.org/ to build *otargen’*s website.

## 3 Usage and documentation

*otargen* can be installed from CRAN running *install.packages(“otargen”)* in the R console. Each *otargen* function features an extensive manual available as function documentation in the R environment using the help flag? *function-name()* in the console. The complete manual with examples can be viewed on the *otargen* website at https://amirfeizi.github.io/otargen/. A separate examples page is available at the “*Get started”* tab of the website, which includes multiple use cases. We recommend using *otargen* for medium-scale queries on short-listed genes or variants to avoid potential performance issues. Open Target Genetics provides alternative approaches for bulk downloading the back-end data designed to accommodate such requirements. For detailed information on data download procedures, please refer to their documentation at https://genetics-docs.opentargets.org/data-access/.

## 4 Conclusion

Identifying the right target is crucial in drug development. Genetic evidence significantly enhances the success rate of drug candidates (Nelson *et al.* 2015, Van Norman 2019). Open Target Genetics is a collaborative effort providing curated data and machine learning tools to prioritize causal variants (Kurki *et al.* 2022). The *otargen* package simplifies tidy data retrieval from OTG’s GraphQL endpoint, facilitating seamless integration of genetics evidence into target discovery pipelines. By streamlining access and analysis of genetic information, *otargen* accelerates the drug development process by facilitating the identification of potential drug targets.

## Data Availability

The data underlying otargen package access are available in at https://genetics-docs.opentargets.org/data-access/data-download.
